# Targeting the DNA damage response enhances CD70 CAR-T cell therapy for renal carcinoma by activating the cGAS-STING pathway

**DOI:** 10.1186/s13045-021-01168-1

**Published:** 2021-09-23

**Authors:** Feng Ji, Fan Zhang, Miaomiao Zhang, Kaili Long, Mingyue Xia, Fei Lu, Enjie Li, Jiannan Chen, Jun Li, Zhengliang Chen, Li Jing, Shaochang Jia, Rong Yang, Zhigang Hu, Zhigang Guo

**Affiliations:** 1 Jiangsu Key Laboratory for Molecular and Medical Biotechnology, College of Life Sciences, Nanjing Normal University, Nanjing, 210023 China; 2 Nanjing Blue Shield Biotechology Co., Ltd., Nanjing, 210023 China; 3 Jinling Hospital of Nanjing University, Nanjing, 210002 China; 4 Department of Urology, Nanjing Drum Tower Hospital, Nanjing, 210008 China

**Keywords:** CAR, CD70, RCC, PARP, Renal carcinoma, Tumor microenvironment, cGAS-STING pathway

## Abstract

Chimeric antigen receptor T-cell (CAR-T) therapy has shown tremendous success in eradicating hematologic malignancies. However, this success has not yet been extrapolated to solid tumors due to the limited infiltration and persistence of CAR-T cells in the tumor microenvironment (TME). In this study, we screened a novel anti-CD70 scFv and generated CD70 CAR-T cells that showed effective antitumor functions against CD70^+^ renal carcinoma cells (RCCs) both in vitro and in vivo. We further evaluated the effect and explored the molecular mechanism of a PARP inhibitor (PARPi) in CAR-T cell immunotherapy by administering the PARPi to mouse xenografts model derived from human RCC cells. Treatment with the PARPi promoted CAR-T cell infiltration by stimulating a chemokine milieu that promoted CAR-T cell recruitment and the modulation of immunosuppression in the TME. Moreover, our data demonstrate that PARPi modulates the TME by activating the cGAS-STING pathway, thereby altering the balance of immunostimulatory signaling and enabling low-dose CAR-T cell treatment to induce effective tumor regression. These data demonstrate the application of CD70 CAR-T cell therapeutic strategies for RCC and the cross-talk between targeting DNA damage responses and antitumor CAR-T cell therapy. These findings provide insight into the mechanisms of PARPis in CAR-T cell therapy for RCC and suggest a promising adjuvant therapeutic strategy for CAR-T cell therapy in solid tumors.

**Supplementary Information:**

The online version contains supplementary material available at 10.1186/s13045-021-01168-1.

## To the editor,

Chimeric antigen receptor T (CAR-T) cell therapy is emerging as a promising treatment and has achieved beneficial effects in cancer patients [1]. However, the application of CAR-T cell therapy for solid tumors (including renal cell carcinoma, RCC) has been hampered by numerous challenges [2]. It was determined that hematologic malignancies and solid tumors, including RCC, can constitutively overexpress CD70, which was shown to be an effective target of CAR-T cells in vitro and in vivo [3]. PARP inhibitors (PARPis), the cancer therapeutic agents targeting poly (ADP-ribose) polymerases (PARPs), which play a key role in the DNA damage repair process, have been reported to increase the recruitment of CD8^+^ T cells to the TME in mouse xenografts [4]. Here, we generated CD70 CAR-T cells with a novel efficient anti-CD70 scFv, and further investigated the effects and mechanisms of PARPi in regulating CAR-T cell therapy in RCC.

**Fig. 1:**
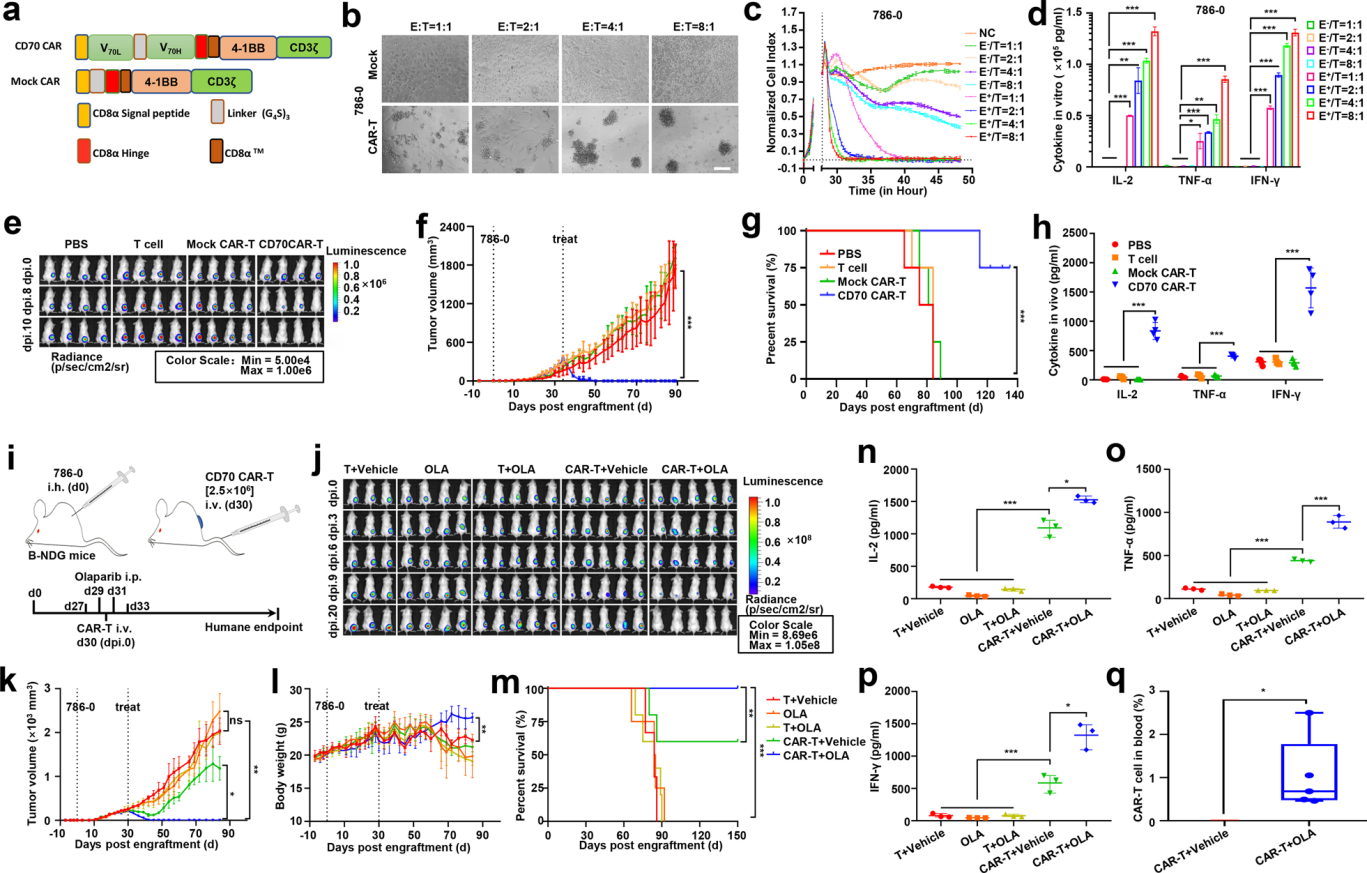
PARP inhibitors promote tumor regression for RCC models under low-dose CD70 CAR-T cell treatment. **a** Schemas of CD70-CARs incorporating different spacers [CD8α Signal peptide, CD8α Hinge, (G_4_S)_3_ Linker, and CD8α™] and costimulatory domains (4-1BB). **b** Lysis of spheres of 786-0 target cell cultures in the presence of CD70 CAR-T cells, or Mock CAR-T cells (control) at a 1:1, 2:1, 4:1, 8:1 effector/target ratio. (Scale bar: 250 μm) **c** A real-time cytotoxicity assay (xCElligence RTCA SP) was used to evaluate the lysis of the indicated tumor cells when treated with mock CAR-T (E^−^) cells or CAR-T (E^+^) cells at the indicated E/T ratios over a 50-h period. Representative of three independent experiments. **d** ELISA results showed the IL-2, TNF-α and IFN-γ secretion levels by CD70 CAR-T (E^+^), mock CAR-T (E^−^) cells encountering 786-0 cell. **e** Subcutaneous renal cell carcinoma tumor (786-0) development was monitored by in vivo bioluminescence imaging (5 × 10^6^ CAR-T cells/mice). Images taken at dpi. 0, 8, 10 are shown (exposure time of 40 s). **f** Data showing the tumor volume (mm^3^) change trend of B-NDG mice xenograft 786-0 tumor regression/growth in 4 different treat groups. **g** Kaplan–Meier survival curve was performed 140 days after 786-0 cells injection. Mice treated with CD70 CAR-T cells had a significantly longer survival probability in comparison with mice treated with PBS, T or mock CAR-T cells. **h** ELISA results showed the IL-2, TNF-α and IFN-γ secretion levels in mice blood treated by PBS, T, mock CAR-T, CD70 CAR-T cells. **i** Treatment scheme used in the 786-0 xenograft model treated with OLA and CAR-T cells. **j** B-NDG mice were treated with OLA and 2.5 × 10^6^ CAR-T cells/mice. Subcutaneous renal cell carcinoma tumor (786-0) development was monitored by in vivo bioluminescence imaging. Images taken at dpi. 0, 3, 6, 9, 20 are shown (exposure time of 40 s). **k** Data showing the tumor volume (mm^3^) change trend of B-NDG mice in 5 different treat groups. **l** Mice body weights monitored during treatment. **m** Kaplan–Meier survival curve was performed 150 days after 786-0 cells injection. Mice treated with CD70 CAR-T cells had a significantly longer survival probability in comparison with mice treated with T + Vehicle (DMSO), olaparib (OLA), T + OLA, CAR-T + Vehicle, CAR-T + OLA. **n**–**p** ELISA results showed the IL-2, TNF-α and IFN-γ secretion levels in mice blood treated by T + Vehicle, OLA, T + OLA, CAR-T + Vehiele, CAR-T + OLA. **q** CD70 CAR-T cells in peripheral blood were detected using flow cytometry on day15 after CAR-T inoculation. All error bars represent SD. In all plots, ns, not significant; *, *p* < 0.05; **, *p* < 0.01; ***, *p* < 0.001

Initially, we confirmed the elevated and specific expression of CD70 in RCC as well as in kidney cancer 786-0, A498, and 769-P cells, which paralleled the poor survival prognosis for renal cancer patients with high CD70 expression (Additional file 1: Fig. S1a–e). This result suggested that CD70 might be an effective target for CAR-T cell immunotherapy in RCC [3]. A second-generation humanized CAR was designed with a novel highly efficient anti-CD70 scFv that was identified from a human scFv phage display library, and the CAR sequence was subcloned into a lentivirus vector (Fig. 1a and Additional file 1: Fig. S2a–d). The percentage of stabilized positive cells was approximately 76% after infection for 12 days, and the defined CD4/CD8 T-cell ratio was 1:1 (Additional file 1: Fig. S2e–h) [5]. The CD70 expression was undetectable in both CD70 CAR-T cells and inactivated T cells, whereas it was detected in the CD3/CD28 antibody-activated T cells (Additional file 1: Fig. S2i, j).

Next, we investigated the antitumor activity of our CD70 CAR-T cells in vitro and in vivo. CD70 CAR-T cells but not mock CAR-T cells induced significantly more target cell lysis of 786-0, A498, and 769-P cells than of HEK293 control cells (Fig. 1b, c and Additional file 1: Fig. S3a, b). Consistently, an increase in cytokines (IL-2, TNF-α, and IFN-γ) was observed in a coculture of CAR-T cells and CD70-positive tumor cells (Fig. 1d and Additional file 1: Fig. S3c). Notably, CD70 CAR-T cells induced a significant lytic effect on HEK293^CD70+^ cells but not on HEK293 cells, highlighting the antigen specificity of the target cell lysis (Additional file 1: Fig. S3d, e). Furthermore, mouse xenografts (derived from 786-0^luc+^ cells) treated with CD70 CAR-T cells showed a significantly decreased RCC burden and exhibited longer survival times than mice treated with PBS, T cells, or mock CAR-T cells (Fig. 1e–g and Additional file 1: Fig. S3f–h). Moreover, higher cytokine (IL-2, TNF-α, and IFN-γ) secretion levels were detected in the peripheral blood of mice treated with CD70 CAR-T cells than in the peripheral blood of mice treated with control vehicles (Fig. 1h). These results demonstrated that our CD70 CAR-T cells were effective in treating CD70^+^ RCCs in vitro and in vivo.

**Fig. 2:**
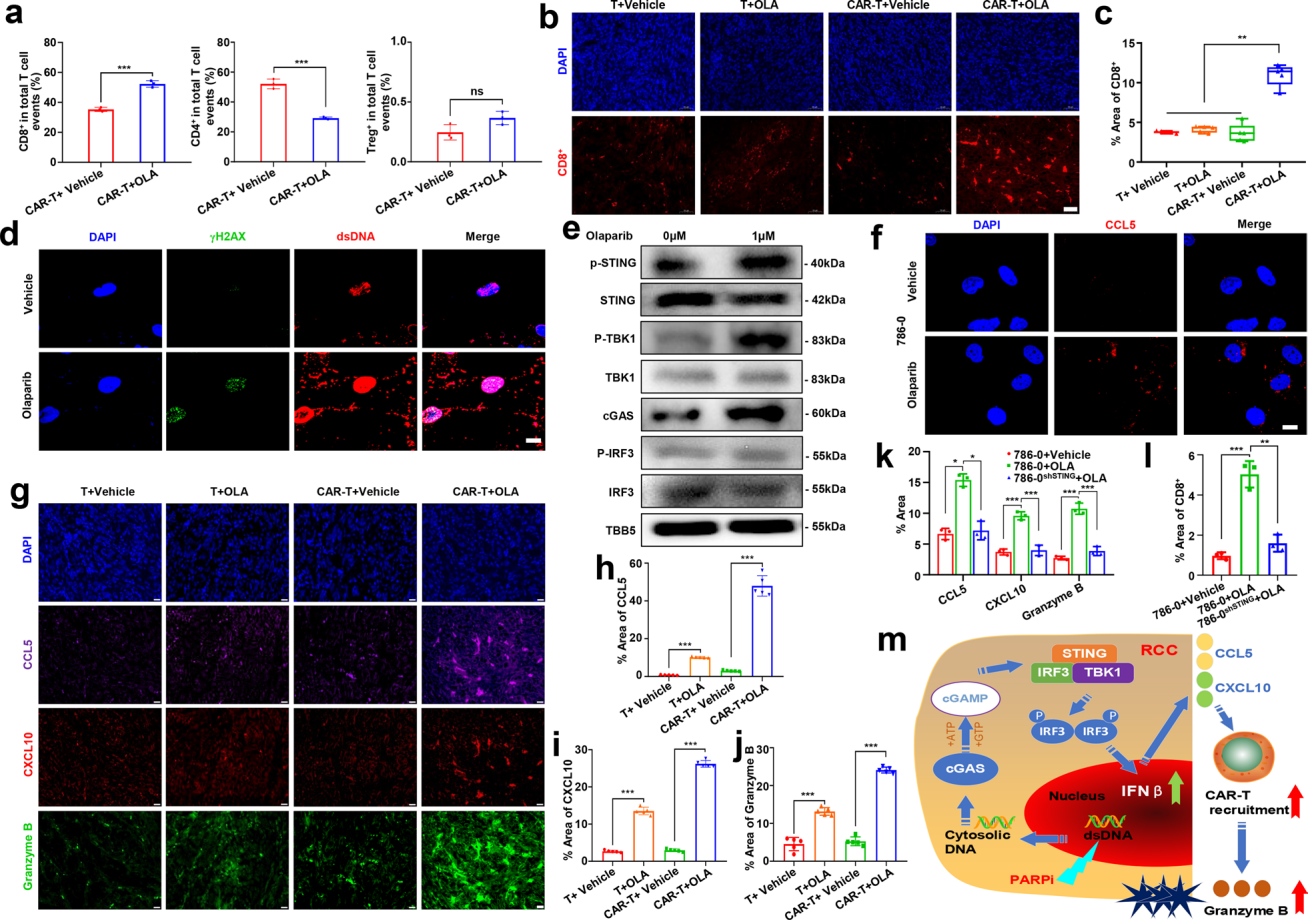
Olaparib activation of cGAS-STING pathway is key to promote tumor killing of CAR-T cell. **a** Olaparib (OLA)- and vehicle (DMSO)-treated tumors were harvested 5 days post-treatment, detecting the phenotype of CAR-T/T cells infiltrating in tumor tissue by flow cytometry. **b** OLA- and DMSO-treated tumors were harvested 5 days post-treatment, subjected to immunofluorescence (IF) analysis for CD8. (Scale bar: 50 μm) **c** Statistical analysis of CD8^+^ T cells in the results of immunofluorescence analysis. **d** Representative images of the level of γH2AX and cytosolic double-stranded DNA (dsDNA) in 786-0 cells after OLA or DMSO treatment. Scale bar, 10 μm. **e** Immunoblots of markers in the cGAS-STING pathway including total and phospho (p) STING (S366), total and phospho TBK1 (S172), cGAS, total and phospho IRF3 (S396) in lysates collected from RCC cell lines treated with OLA or DMSO. TBB5 served as a loading control. **f** Representative images of the level of chemokines CCL5 in 786-0 cells after OLA or DMSO treatment. Scale bar, 10 μm. **g** The CCL5, CXCL10 and Granzyme B IF staining were performed in tumors from the resected tumors from Fig. 2b. Representative images of staining intensity are shown. (Scale bar, 20 μm). **h**–**j** Quantification of tumor sections immunostained for CCL5, CXCL10 and Granzyme B -positive areas quantified for each field (*N* = 5). **k** CAR-T- and OLA-treated 786-0^shSTING^ tumors were harvested 5 days post-treatment, quantification of CCL5, CXCL10 and Granzyme B in the results of IF analysis from Additional file 1: Fig. S6f (*N* = 3). **l** CAR-T- and OLA-treated 786-0^shSTING^ tumors were harvested 5 days post-treatment, statistical analysis of CD8^+^ T cells in the results of IF analysis from Additional file 1: Fig. S6g (*N* = 3). **m** Model for cGAS-STING pathway activation in response to DDR targeting in RCC. In the proposed model, targeting the DDR protein PARP with the small-molecule inhibitor OLA leads to cytosolic DNA in RCC models. The cytosolic DNA is then recognized by cGAS, which leads to activation of the STING/TBK1/IRF3 pathway. IRF activation leads to increased expression of IFNβ and enhanced expression of the chemokines CXCL10 and CCL5. STING pathway activation and increased chemokine expression lead to the recruitment and secretion of large amounts of Granzyme B by CD8^+^ CAR-T cells leads to enhanced antitumor immunity in RCC models. All error bars represent SD. In all plots, ns, not significant; *, *p* < 0.05; **, *p* < 0.01; ***, *p* < 0.001

The PARPi olaparib (OLA) has been shown to improve the antitumor efficacy of CAR-T cells in breast cancer [6]. We further evaluated the effect of OLA in CAR-T cell therapy in RCC. We first explored the impact of OLA on CAR-T cells and found that OLA showed little effect on CAR-T cell viability when the OLA concentration was lower than 2.5 μM (Additional file 1: Fig. S4a, b). We then pretreated RCC cells with OLA following CAR-T cells incubation to detect the effect of OLA on different RCC cells. Cell apoptosis assays indicated that OLA pretreatment promoted the apoptosis of RCC cells but protected CAR-T cells against apoptosis (Additional file 1: Fig. S4c, d). Then, we designed an experimental scheme using mouse xenograft models treated with OLA and CAR-T cells (Fig. 1i). Notably, compared with the control treatments, the combinational treatment of OLA and CD70 CAR-T cells (CAR-T + OLA) showed more effective repression of RCC xenografts, which was accompanied by a better survival rate among tumor-bearing mice (Fig. 1j–m). The serum levels of TNF-α, IL-2, and IFN-γ were also higher in the CAR-T + OLA group than in the other four groups (Fig. 1n–p). Notably, CAR-T cells were observed on day 15 post CAR-T injection (dpi. 15) in the blood of the CAR-T + OLA group but not in the CAR-T + Vehicle groups (Fig. 1q), which further supported that OLA treatment promoted CAR-T cell persistence. Moreover, multicolor flow cytometry and IHC assays showed that OLA treatment increased the infiltration of CD8^+^ CAR-T cells but not CD4^+^ CAR-T cells and Treg cells in the TME 5 days after injection of CAR-T cells (Fig. 2a–c and Additional file 1: Fig. S5a, b).

Finally, to determine the molecular mechanism of OLA-induced cytotoxic CAR-T cell recruitment in the TME, we detected the activation of the cGAS/STING signaling pathway, which has been reported to be a critical activator in antitumor immune responses and in CD8^+^ T-cell recruitment with PARPi treatment [7, 8]. Elevated accumulation of cytosolic DNA and the DNA damage marker γH2AX was detected in 786-0 cells after treatment with OLA (Fig. 2d). The sensors and key regulators in the cGAS/STING pathway, including cGAS, and the phosphorylation of STING, TBK1, and IRF3, were all upregulated in cells treated with OLA, which was accompanied by upregulation of IFN-β expression (Fig. 2e and Additional file 1: Fig. S5c, d) [9]. The upregulation of IFN-β paralleled the observed increases in chemokines such as CCL5 and CXCL10, which are key mediators of the chemotaxis of CD8^+^ T lymphocytes (Fig. 2f–i and Additional file 1: Fig. S5e, f) [9, 10]. The increase in CD8^+^ CAR-T cells ultimately enhanced tumor lysis because of their secretion of large amounts of granzyme B (Fig. 2g, j) [11]. Moreover, knockdown of STING impaired the OLA-induced CCL5 and CXCL10 production in 786-0 cells (786-0^shSTING^) as well as in tumor tissues derived from 786-0^shSTING^ cells, indicating that PARP inhibitor–induced proinflammatory cytokine production is mediated through the cGAS-STING pathway (Fig. 2k and Additional file 1: Fig. S6a–f). Notably, OLA-induced CD8^+^ CAR-T cell recruitment and granzyme B expression was abolished in the 786-0^shSTING^ tumor tissues (Fig. 2k, l and Additional file 1: Fig. S6f, g). These data demonstrated that OLA-mediated CAR-T infiltration and persistence in TME are dependent on the cGAS-STING pathway [12].

In summary, we demonstrated the efficacy of CD70 CAR-T cells in RCC immunotherapy, and PARPi treatment enhanced this immunotherapy by promoting the infiltration of CD8^+^ CAR-T cells in the TME by activating the cGAS/STING signaling pathway (Fig. 2m). This study indicates that the combination of CAR-T cell therapy with PARPi represents a potential therapeutic approach for solid tumors.

## Supplementary Information

**Additional file 1**: Supplementary Figures.

**Additional file 2**: Supplementary materials and methods.

## Data Availability

The authors declare that all data supporting the results in this study are available within the paper and its supplementary information. Source data for the figures in this study are available from the corresponding author upon reasonable request.

## Abbreviations

CAR-T: Chimeric antigen receptor T-cell
TME: Tumor microenvironment
PARP: Poly ADP-ribose polymerase
RCC: Renal cell carcinoma
PARPi: PARP inhibitors
DC: Dendritic cells
OLA: Olaparib
PBMCs: Peripheral blood mononuclear cells
GFP: Green fluorescent protein
scFv: Single-chain variable fragment
