# Supplementary material for: Targeting the DNA damage response enhances CD70 CAR-T cell therapy for renal carcinoma by activating the cGAS-STING pathway
Source: J Hematol Oncol. 2021 Sep 23;14:152. doi: 10.1186/s13045-021-01168-1 (PMC8461872; doi:10.1186/s13045-021-01168-1)
Supplement: Supplementary file 1 — Additional file 1: Supplementary Figures. [file 13045_2021_1168_MOESM1_ESM.docx]

**Additional file 1.**


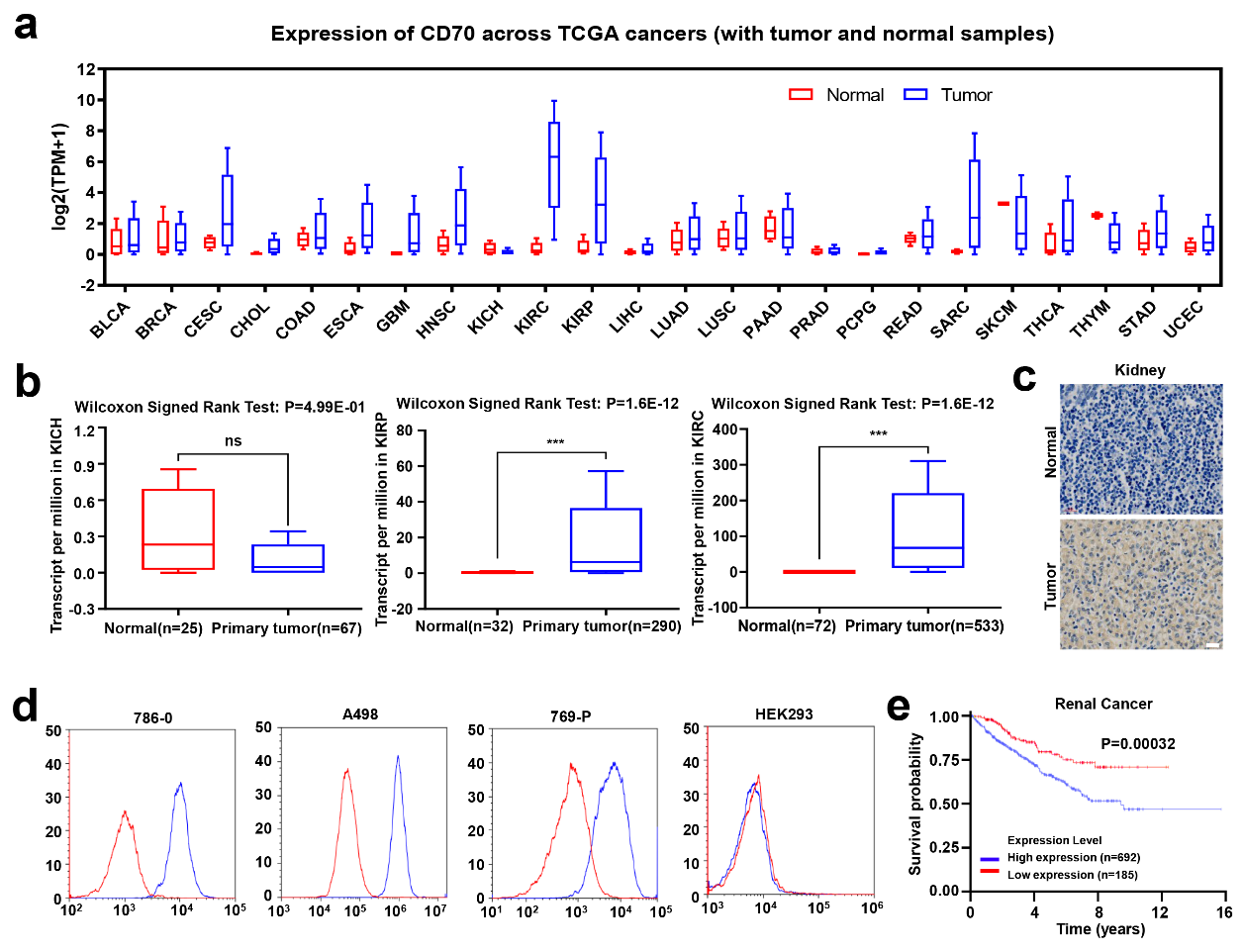


**Fig. S1:** Analysis of CD70 expression in human normal tissue, primary renal cell carcinoma tissues and cell lines. **a** Differential expression profile analysis of CD70 in tumor and normal tissues based on the TCGA database. **b** Normalized mRNA levels of CD70 in tumor and normal tissues using the online web server GEPIA. KICH, Kidney Chromophobe; KIRP, Kidney renal papillary cell carcinoma; KIRC, Kidney renal clear cell carcinoma. **c** Microarrays of human Kidney were stained for IHC to detect the expression of CD70. Scale bars, 20 μm. **d** Expression of CD70 in human cell lines were evaluated by FACS. Cells were incubated with Anti-CD70 antibody (ab223292, blue) or its corresponding isotype control (red). **e** Kaplan-Meier plots summarize results from analysis of correlation between mRNA expression level and patient survival (https://www.proteinatlas.org). Patients were divided based on level of expression into one of the two groups "low" (under cut off) or "high" (over cut off). X-axis shows time for survival (years) and y-axis shows the probability of survival, where 1.0 corresponds to 100 percent. In all plots, ns, not significant; *, p < 0.05; **, p< 0.01; ***, p < 0.001.


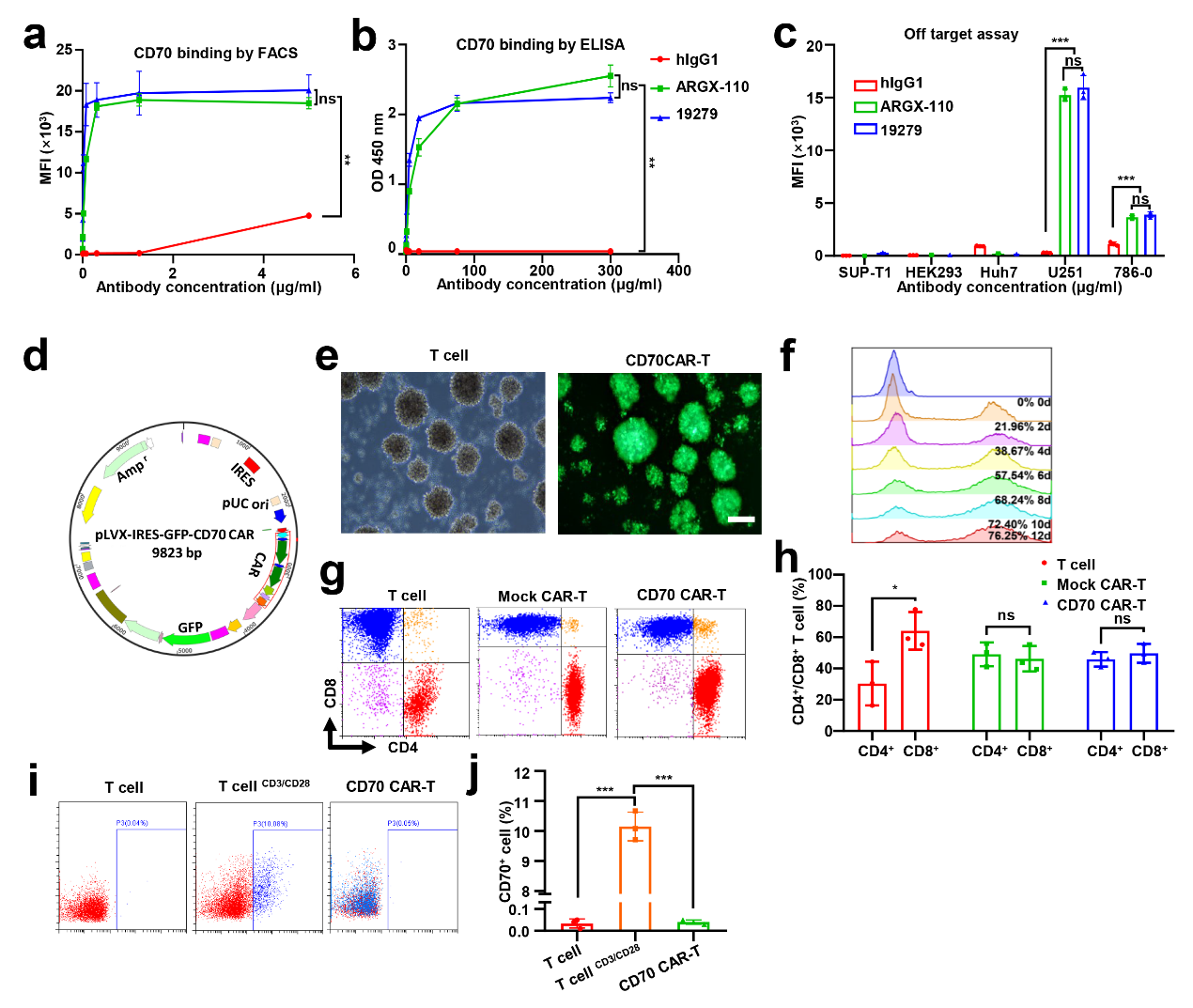


**Fig. S2:** Construction and characterization of CD70-specific CARs. **a** Screening of anti-CD70 antibodies (19279) by FACS. ARGX-110 as positive control, hIgG1 as negative control. **b** Binding of 19279 in an ELISA to human CD70 Protein (P32970-1, ACRO) equivalent compared with the ARGX-110. **c** Off target assay of 19279 to CD70-positive cell lines (786-0, U251) and the CD70 negative cell lines (Huh-7, HEK293 and SUP-T1) as determined by flow cytometry. Data presented as mean of triplicate measurements. **d** The lentiviral backbone plasmid encodes the CD70 CAR. **e** Images of transduced CAR-T cells were captured using inverted fluorescent microscope. (Scale bar: 250 μm) **f** The transduction efficiency was measured by GFP positive cells using flow cytometric analysis. **g** Efficient lentiviral transduction of primary human T cells encoding anti-CD70 CAR, with similar CD4 / CD8 ratios in control and CAR transduced T cells. (12 days after lentivirus CAR transduction, the subsets and phenotype of T cell, Mock CAR-T and CD70 CAR-T cells were analyzed by FACS, including the expression of CD4 and CD8.) **h** CD4 / CD8 statistics results. **i, j** The expression levels of CD70 on the surface of inactivated/ activated T cells, and generated CD70 CAR-T cells were detected by flow cytometry. All error bars represent SD. In all plots, ns, not significant; *, p < 0.05; **, p< 0.01; ***, p < 0.001.


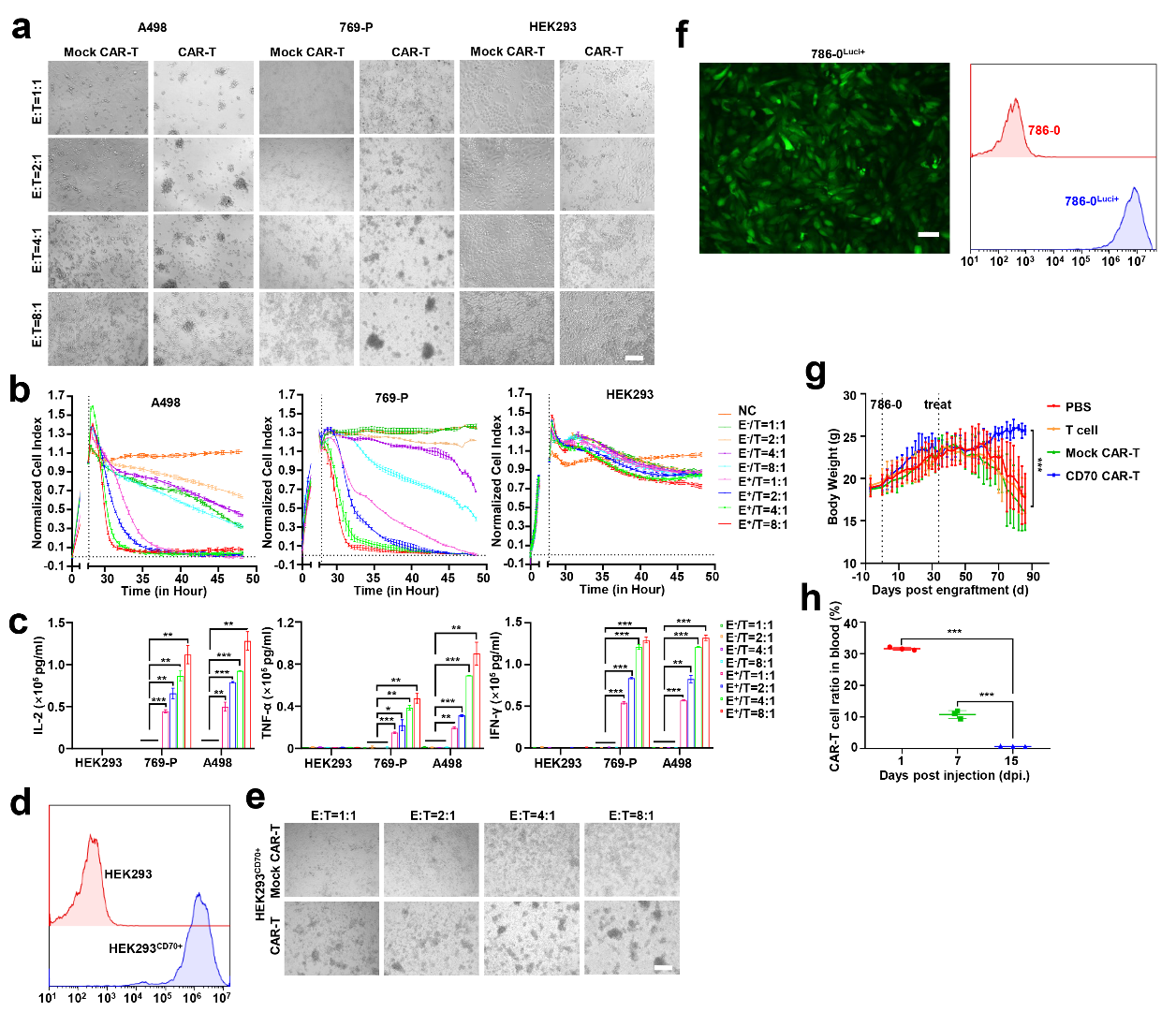


**Fig. S3:** Cytotoxicity activities and cytokine production of CD70-specific T cells *in vitro*. **a** Lysis of spheres of A498, 769-P and HEK293 target cell cultures in the presence of CD70 CAR-T cells, or Mock CAR-T cells (control) at a 1:1, 2:1, 4:1, 8:1 effector: target ratio. Effector cells from three different donors were used with consistent results. (Scale bar: 250 μm) **b** A real-time cytotoxicity assay (xCElligence RTCA SP) was used to evaluate the lysis of the indicated tumor cells when treated with mock CAR-T (E^-^) cells or CAR-T (E^+^) cells at the indicated E:T ratios over a 50-hour period. Representative of three independent experiments. **c** ELISA results showed the IL-2, TNF-α and IFN-γ secretion levels by CD70 CAR-T (E^+^), Mock CAR-T (E^-^) cells encountering A498, 769-P or HEK293 cell lines. **d** Lentivirus-mediated CD70 overexpression in HEK293 cell line by flow cytometry (blue is the peak of overexpression cells). **e** Lysis of spheres of HEK293^CD70+^ target cell cultures in the presence of CD70 CAR-T cells, or Mock CAR-T cells at a 1:1, 2:1, 4:1, 8:1 effector: target ratio. Effector cells from three different donors were used with consistent results. (Scale bar: 250 μm) **f** Renal cancer cell line 786-0 overexpressing luciferase, which was used to construct a mouse subcutaneous transplantation tumor model, was detected by flow cytometry and fluorescence microscopy. **g** Body weights were measured before tumor injection, before CAR-T cell injection, and tested three times a week after CAR-T cell (5×10^6^ CAR-T cells/mice) injection and compared with untreated B-NDG mice. **h** CD70 CAR-T cells in peripheral blood were detected using flow cytometry on day 1, 7, 15 post CAR-T (5×10^6^ CAR-T cells/mice) injection. All error bars represent SD. In all plots, ns, not significant; *, p < 0.05; **, p< 0.01; ***, p < 0.001.


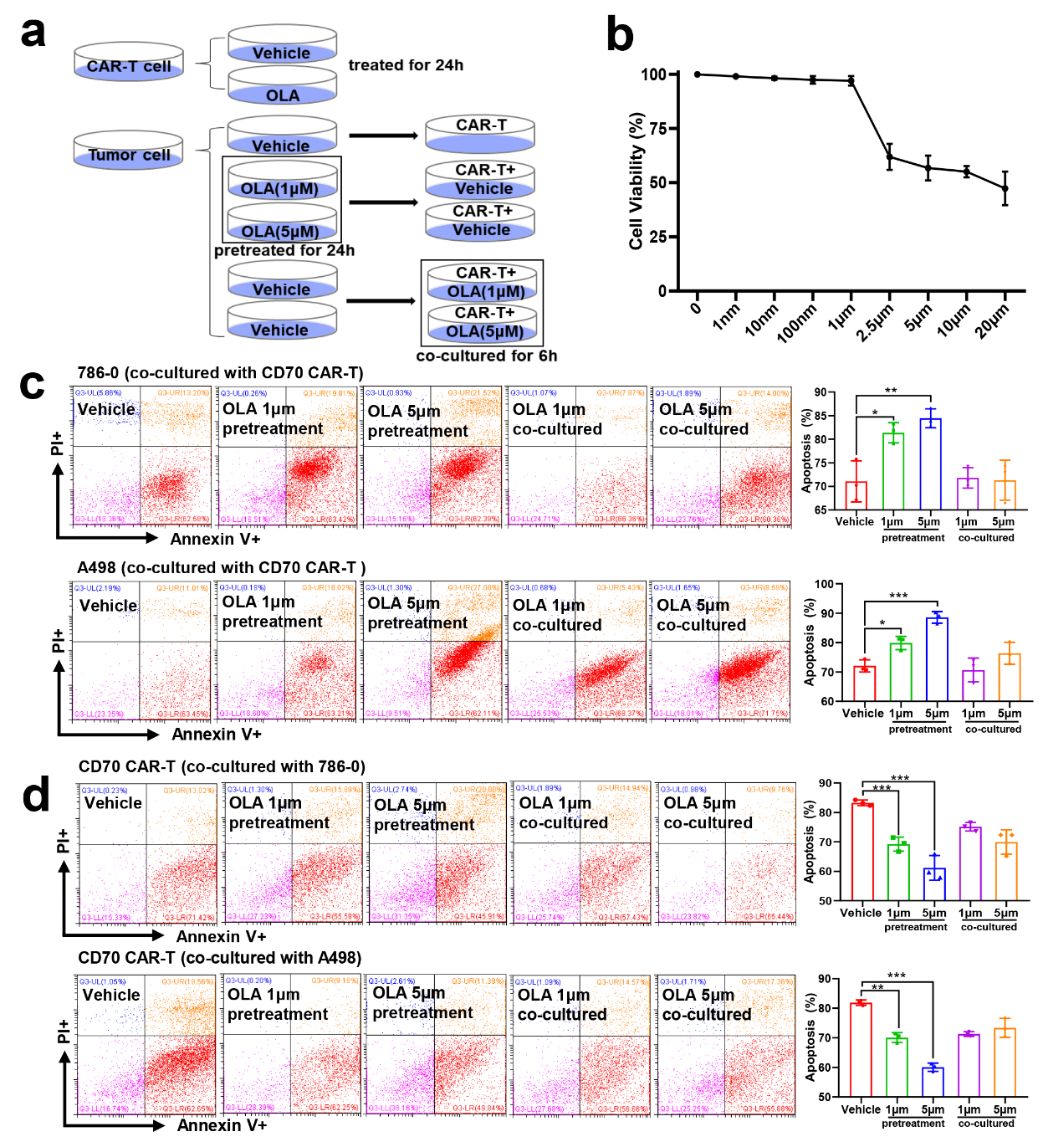


**Fig. S4:** Effects of olaparib (OLA) on CAR-T cells and RCC cells *in vitro*. **a** Schematic diagram of cytotoxic activities of CAR-T cells combined with OLA. **b** The cytotoxicity of OLA to CAR-T cells was shown after treatment with indicated concentrations of OLA for 24 h. **c, d** Operating procedure according to Fig. S4a, tumor cells that pretreatment for 24h with OLA or not was incubated with CAR-T cells at an E/T ratio of 1:1 in 48-well plates, and CAR-T cells were separated from tumor cells after co-culture at 37°C for 6 h. The representative flow cytometry plots showing the apoptosis frequencies of tumor cells (**c**) and CAR-T cells (**d**). All error bars represent SD. In all plots, *, p < 0.05; **, p< 0.01; ***, p < 0.001.


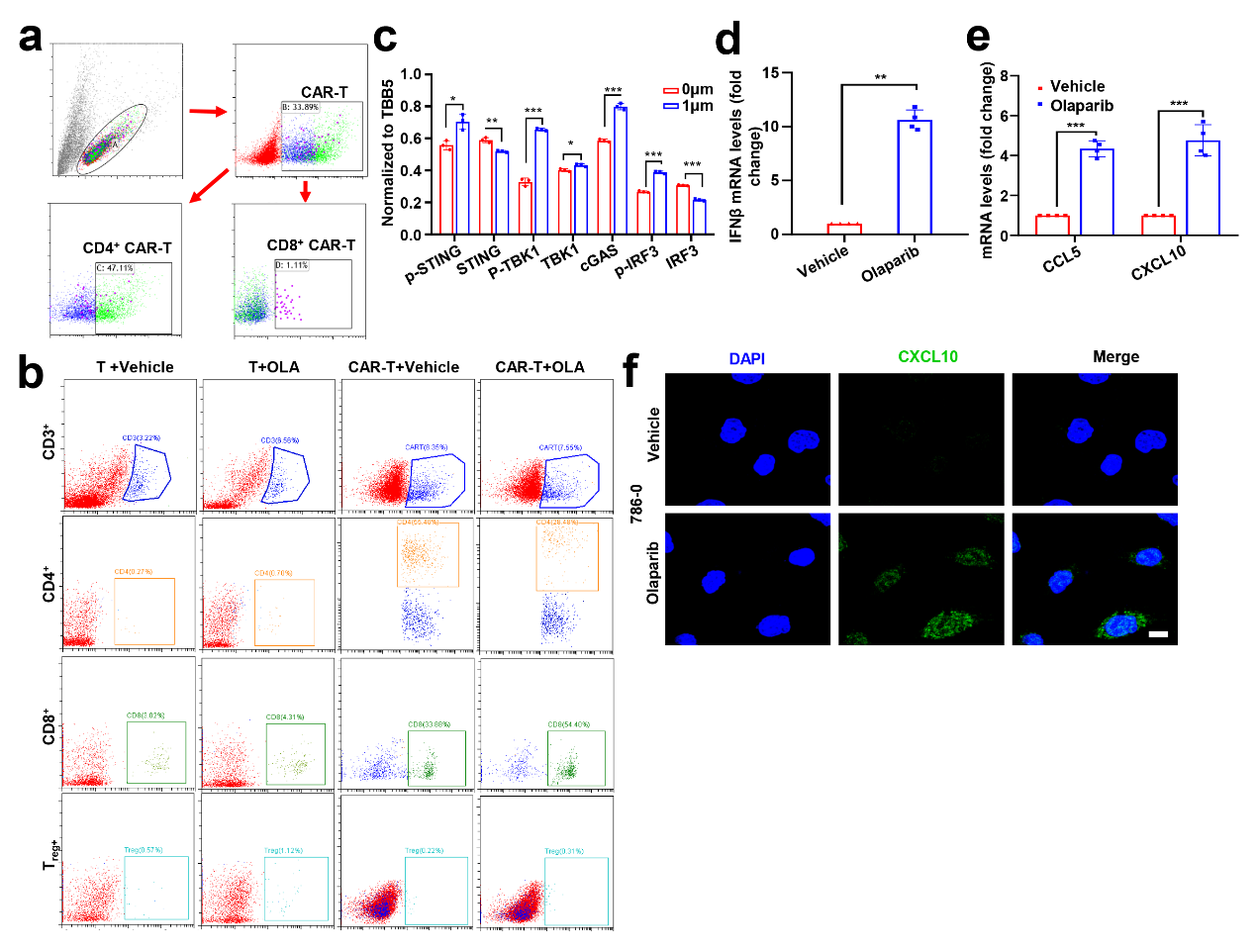


**Fig. S5:** PARP inhibitors promote tumor regression for RCC models under low-dose CD70 CAR-T treatment. **a** The phenotype analysis strategy of CAR-T cells in tumor tissue of mice after CAR-T treatment of renal carcinoma subcutaneous xenograft tumor model. **b** OLA or vehicle (DMSO)-treated tumors were harvested 5 days post-treatment, detect the phenotype of CAR-T / T cells infiltrating in tumor tissue by flow cytometry. **c** Quantification of markers in the cGAS-STING pathway including total and phospho (p) STING (S366), total and phospho TBK1 (S172), cGAS, total and phospho IRF3 (S396) in lysates collected from RCC cell lines treated with OLA. **d** qPCR analysis of IFNβ mRNA levels in OLA or DMSO treated 786-0 for 72 hours, normalized to GAPDH internal control. **e** qPCR analysis of CCL5 and CXCL10 mRNA in 786-0 cell line after OLA treatment for 72 hours, normalized to GAPDH internal control. **f** Representative image of the level of chemokines CXCL10 in 786-0 cells after OLA or vehicle (DMSO) treatment. (Scale bar, 10 μm). In all plots, ns, not significant; *, p < 0.05; **, p< 0.01; ***, p < 0.001.


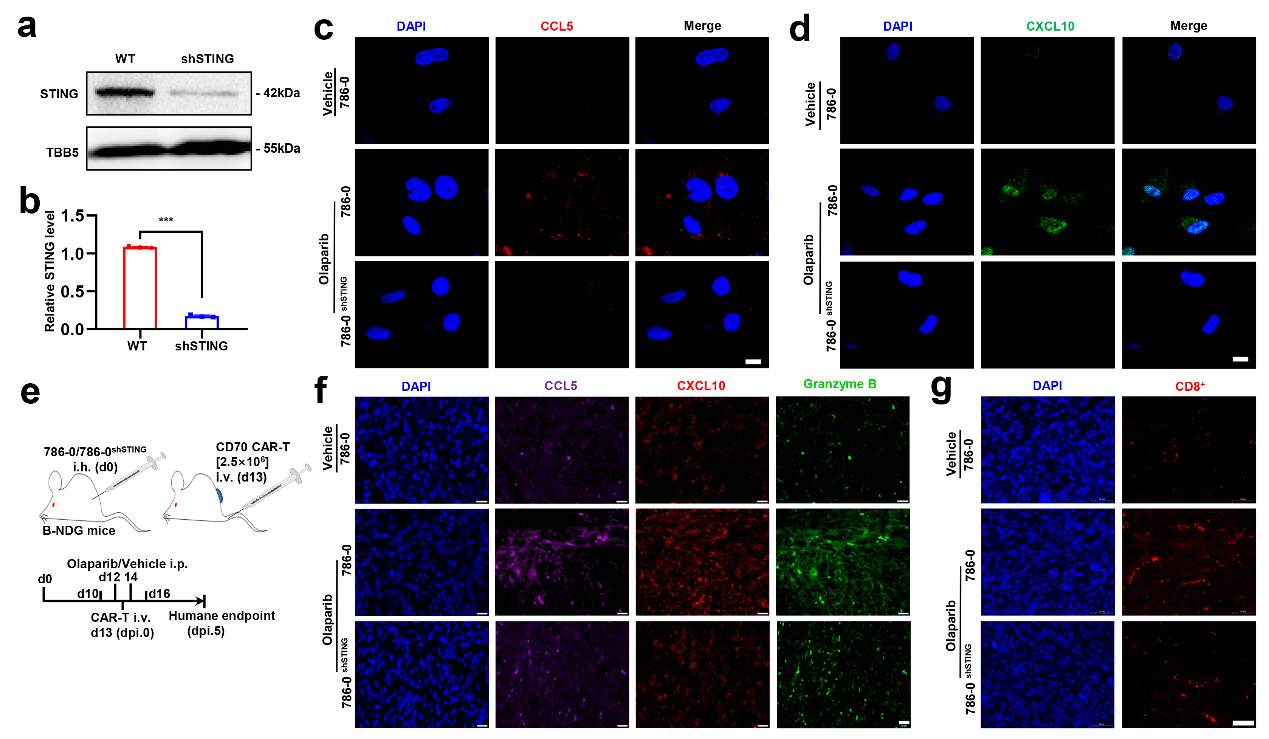


**Fig. S6:** STING depletion abolishes olaparib-induced proinflammatory signaling and CAR-T cells infiltration in TME. **a** Western blot for total STING protein levels in 786-0 WT or STING knock-down cells. **b** Quantification of relative STING levels shown in (**a**), with three independent assays. **c, d** Representative image of the level of chemokines CCL5 (**c**) and CXCL10 (**d**) in 786-0^shSTING^ cells after OLA or vehicle (DMSO) treatment. (Scale bar, 10 μm). **e** Treatment scheme used in the 786-0^shSTING^ xenograft model treated with OLA and CAR-T cells. **f** The CCL5, CXCL10 and Granzyme B immunofluorescence (IF) staining were performed in tumors from the resected 786-0^shSTING^ tumors after Fig. S6e treated. Representative images of staining intensity are shown. (Scale bar, 20 μm). **g** CAR-T and OLA-treated 786-0^shSTING^ tumors were harvested 5 days post-treatment, subjected to IF analysis for CD8. (Scale bar: 50 μm) In all plots, ns, not significant; *, p < 0.05; **, p< 0.01; ***, p < 0.001.
