# Supplementary material for: Targeting the DNA damage response enhances CD70 CAR-T cell therapy for renal carcinoma by activating the cGAS-STING pathway
Source: J Hematol Oncol. 2021 Sep 23;14:152. doi: 10.1186/s13045-021-01168-1 (PMC8461872; doi:10.1186/s13045-021-01168-1)
Supplement: Supplementary file 2 — Additional file 2: Supplementary materials and methods. [file 13045_2021_1168_MOESM2_ESM.docx]

**Additional file 2.**

**Targeting the DNA damage response enhances CD70 CAR-T therapy for renal carcinoma by activating the cGAS-STING pathway**

Feng Ji^1^, Fan Zhang^1^, Miaomiao Zhang^1^, Kaili Long^1^, Mingyue Xia^1^, Fei Lu^1^, Enjie Li^1^, Jiannan Chen^1^, Jun Li^2^, Zhengliang Chen^2^, Li Jing^2^, Shaochang Jia^3^, Rong Yang^4^, Zhigang Hu^1*^, Zhigang Guo^1*^

**Affiliations**

^1^ Jiangsu Key Laboratory for Molecular and Medical Biotechnology, College of Life Sciences, Nanjing Normal University, Nanjing, China 210023.

^2^ Nanjing Blue Shield Pharma, Nanjing, China 210023.

^3^ Jinlin Hospital of Nanjing University, Nanjing, China 210002.

^4^ Department of Urology, Nanjing Drum Tower Hospital, Nanjing, China 210008.

**Supplementary Information**

The following file contains supplementary material for the paper “Targeting the DNA damage response enhances CD70 CAR-T therapy for renal carcinoma by activating the cGAS-STING pathway”.

This file is composed of:

Supplementary materials, methods, and references

**Materials and Methods**

Mice

Six-week-old female B-NDG (NOD-*Prkdc^scid^ IL2rg^tm1^*/Bcgen) mice were purchased from Biocytogen (Beijing). Mice were maintained under specific pathogen-free conditions, and all procedures met the Nanjing Normal University Animal Faculty (IRB#2020-0047).

Cell lines

Peripheral blood mononuclear cells (PBMCs, TPCS#PB025C) were purchased from the miles-bio of Shanghai. Glioblastoma U251, Human hepatocellular carcinoma Huh-7, T cell lymphoblastic lymphoma SUP-T1, Normal human embryonic kidney cell line HEK293 and human renal cell adenocarcinoma cell lines 786-0, A489, and 769-P were purchased from the American Type Culture Collection. All cells were cultured in recommended medium supplemented with 10% FBS in a 10% CO_2_ incubator. T lymphocytes were maintained in T cell growth medium (TCGM): X-VIVO 15 Serum-free Hematopoietic Cell Medium supplemented with 5% FBS 2 ng mL^-1^ human recombinant IL-2 (50 U mL^-1^, Sigma, Germany). All cell lines were tested and authenticated by Short Tandem Repeat Profiling (DNA Fingerprinting) within 6 months of the study and routinely tested for Mycoplasma species before any experiment was performed.

Retroviral vector construction

The amino acid sequence of the human CD70 antibody was screened previously in the laboratory [1]. CD8α Signal peptide-anti CD70 single-chain variable fragment (scFv)- CD8α Hinge-CD8^TM^ was created, the hinge region of human 4-1BB, and the human CD3ζ chain were reorganized using standard molecular biology techniques and codon optimization of the complete CAR sequence using GenSmart™ technology. CAR (CD70) was designed and fully synthesized according to the structure of the second-generation CAR. These plasmids were linearized with EcoRI/BamH I and subcloned into a pMDL based expression vector containing the GFP gene as a tracker for detecting the expression of CAR with FCM.

Retrovirus production

293T cells were grown to 80% confluence in T175 Angled Neck Cell Culture Flask with Vent Cap (Corning, USA) and underwent a tripartite transfection with the following plasmids using Polyethyleneimine Linear, MW 40000 (PEI 40K): 18.75 μg of pMD-gag-pol, 6.25 μg of pMD-VSVG, and 25 μg of either pMDL-CD70-CAR. Viral supernatant was collected at 48 h after DNA addition, passed through a 0.22 μm filter (Millex), and refrigerated. Concentrated viral stocks were prepared by centrifugation of viral supernatant in an SW28 rotor at 25,000 ×g, 4 °C, for 2 h. The supernatant was decanted, and 200 μL of TNE solutuib (50 mM Tris, pH 7.4, 30 mM NaCl, 1 mM EDTA, pH 8.0) was added. After an overnight incubation at 4 °C, the virus was resuspended and stored at -80 °C.

**T cell isolation and retroviral transduction**

T cells were isolated from PBMCs by negative selection using EasySep^TM^ Human T Cell Isolation Kit (Stemcell, Canada). Primary lymphocytes were stimulated with anti-CD3/CD28 Dynabeads (Miltenyi, Germany). Detection of the CAR-T cell positive rate and detection of cell phenotype was performed following lentivirus infection and continuous culture for 48 hours after T cell isolation. Cells were allowed to expand in culture until day 15 or 20. For all experiments using CD70 CAR-T cells, paired (from same donor) untransduced T cells, activated and cultured for equivalent time, served as control T cells or Mock CAR-Ts.

**Generation of CAR-modified T cells and gene-modified cell lines**

To generate CAR-T cells, T cells sorted in the previous step were adjusted to 1.0×10^6^ cells mL^-1^ and plated in each well of a non-tissue culture-treated 24-well plate. T cells were cultured in 5% FBS X-VIVO 15 Serum-free Hematopoietic Cell Medium (Lonza, Switzerland), which was supplemented with recombinant human IL-2 and CD3/CD28-microspheres (45 μL per 1.0×10^7^ T cells) were added on day 0. On day 2, cultures were centrifuged at 400 ×g for 5 min to discard the culture medium and collect T cells. T cells were resuspended with 100 μL of CD70 CAR-Ts lentivirus and inoculated in 96-well plates for 4 hours to incubate, and then were re-inoculated into 24-well plates and transferred to a 37 °C, 5% CO_2_ incubator. On day 3 post transduction, T cells were harvested, washed, and cultured in X-VIVO 15 Serum-free Hematopoietic Cell Medium. Cultures were supplemented with fresh medium and IL-2 every 2 days. Transduction efficiency was measured 5–7 days post transduction by flow cytometry. To track the number of T cells over time, viable cells were manually counted using trypan blue. While T cells were generated in X-VIVO 15 media, all *in vitro* functional assays were performed in mixed medium [50% DMEM+50% X-VIVO 15] supplemented with 10% FBS.

**Construction of CD70 overexpressing cell line**

The overexpression cell line was constructed by infection with lentiviral vector, cultured under puromycin conditions to obtain a stable transgenic strain, and the CD70 expression rate was detected by flow cytometry.

**Flow cytometry**

For flow cytometry analyses of human cells and RCC cell lines, cells were stained with mouse anti-human CD70-APC (Abcam, Grand Island, NY), mouse anti-human CD3-FITC (BD, San Diego, CA), goat anti-human IgG- Fcγ fragment specific-APC (BD, San Diego, CA), mouse anti-human CD4-PE (BD, San Diego, CA), mouse anti-human CD8-APC (BD, San Diego, CA), mouse anti-human Foxp3-APC (BD, San Diego, CA), monoclonal antibodies at 1:100 dilution. Expression of CAR proteins was detected using Biotinylated Recombinant Protein L, His Tag, a primary amine labeling, long spacer (Acro, Delaware). Cells were stained with antibodies for 30 min at room temperature. All samples were acquired on a CytoFLEX S (Beckman Coulter, Indianapolis, IN), and data was analyzed using Kaluza 2.1 Flow Analysis Software (Beckman Coulter Life Sciences).

***In Vitro* Cytotoxicity**

Using the impedance-based Real Time Cell Analysis, RTCA (ACEA, San Diego, CA), the kinetics of tumor cell lysis was evaluated over 140 h. 786-0, A498, 769-P tumor cells and HEK293 were plated in a 96-well, resistor-bottomed plate at 2.0×10^4^ cells per well in triplicate. After 24 h, effector T cells were added into the unit at various effector (T cells, Mock CAR-T, CD70 CAR-T)/target cell (E: T) ratios (8:1, 4:1, 2:1, and 1:1). Impedance was measured at 15-min intervals. The impedance-based cell index for each well and timepoint were normalized with the cell index prior to the addition of T cells. Kinetics of tumor cell lysis were assessed as change in the normalized cell index over time. The mean percentage of specific lysis was obtained from experiments performed in triplicate wells. For the toxicity assay of OLA on CAR-T cells, CAR-T cells were treated with different concentrations of OLA in 96-well plates at 37°C for 24 hours. For the study of the effect of OLA on tumor cell killing by CAR-T cells, the medium was pretreated in target cells with or without OLA (1 μM or 5 μM). Briefly, 786-0/ A498 was incubated with CAR-T cells at an E/T ratio of 1:1 in 48-well plates, and CAR-T cells were isolated from tumor cells after co-culture at 37°C for 6 h. According to the manufacturer's protocol, using the Annexin V-Alexa Fluor 647/PI Apoptosis Detection Kit (YEASEN, Shanghai, China) to detect the level of apoptosis in the target cells by flow cytometry.

***In vivo* effectiveness study in B-NDG mice**

B-NDG mice were housed and manipulated in strict accordance with the ethical review committees of the Nanjing Normal University. On day 0, tumor cells were implanted into the subcutaneous space on the right back of recipient mice. Tumor values were measured with calipers three times a week and tumor volumes (in mm^3^) were determined using the formula W^2^L/2, where W is tumor width and L is tumor length. On the days of treatment (tumor volume of 200 mm^3^), T cell, Mock CAR-T and CD70 CAR-T cell (15 days post transduction) were harvested, washed, resuspended in cold PBS, and injected in a volume of 100 μL (5.0×10^6^ CAR-T cells) into the tail vein. Tumor and distribution were evaluated by injecting mice intraperitoneally with 100 μL of D-luciferin (15 mg mL^-1^, PerkinElmer, Waltham, MA) followed by BLI using an IVIS Lumina II imaging system (Caliper Life Sciences, Hopkinton, MA), and analyzed by Living Image software (Caliper Life Sciences, Hopkinton, MA). All mice were euthanized when the negative control reached a mean tumor volume of 2000 mm^3^ or had extensive tumor ulceration. At the end of the intravenous CAR-T cell administration, 100 μL of blood was collected and stained with different antibodies by flow cytometry as indicated above.

**shRNA-Mediated STING Knockout**

The short hairpin RNA (shRNA) sequences 5’- CAACATTCGATTCCGAGATAT -3’ targeting human STING was cloned into the pGpU6-GFP-Neo vector. For generation of 786-0 STING knock-down cells were transfected with pGpU6-GFP-Neo using lipofectamine 3000 transfection reagent (Invitrogen). Plasmid-incorporated cells were enriched by geneticin selection (500 μg/mL; Merck, Germany) for 2 weeks, and polyclonal populations were used in experiments. Tumor generation was performed as stated above for the 786-0 model.

**Quantification of cytokines**

All co-culture experiments were performed with a CD4^+^ cell-to-CD8^+^ cell ratio of 1, with 50% CAR^+^ population. Tumor cells (2×10^4^) were inoculated in 96-well plates. After 24 hours, T cells, Mock CAR-T, CD70 CAR-T were added at an effector-to-target ratio of 1:1, 2:1, 4:1, 8:1. For cytokine production assays, supernatants were collected 12 hours after co-culture and assessed for human IL-2, TNF-α, IFN-γ levels using the SenBeiJia Biological Enzyme‐Linked Immunosorbent Assay (ELISA) Development Kit, as per the manufacturer’s protocol. Blood was left at room temperature for 20 min to clot, then centrifuged to obtain serum. Each experiment was carried out independently in triplicate.

**The effect of PARP inhibitors on tumor microenvironment in CAR-T immunotherapy**

OLA (AZD2281, Selleck) was reconstituted in Vehicle (DMSO) (12.5 mg mL^-1^). The dosage is calculated as 50 mg/kg body weight. B-NDG mice formed tumors according to the above method and were divided into five groups (T+ Vehicle, OLA, T+OLA, CAR-T+Vehicle, CAR-T+OLA). Mice were given corresponding treatment when the tumor size was between 200–300 mm^3^. Mice (T+Vehicle, T+OLA, CAR-T+Vehicle, CAR-T+OLA, 5 per group) were sacrificed on the day 5 after treatment. Tumors were extracted, finely minced, blended with gentle Collagenase Ⅳ (Solarbio, Beijing, China), and digested in a 37 °C CO_2_ incubator for 30 min according to the manufacturer’s instructions. Dissociated tumor cells were washed with RPMI-1640 medium (Gibco, USA). Cells were resuspended in FCM buffer: PBS (GE Life Technologies) containing 0.5% BSA and 2 mmol L^-1^ EDTA (Sigma, Germany). The method indicated above was used to stain the cells to detect the T cells phenotype that migrated into the tumor tissue, and were analyzed using a CytoFLEX S flow cytometer. Compensation was performed manually on CytExpert using single color and isotype controls. Signal threshold definition was defined using all-stain, unstained, and isotype controls. Analysis was performed using Kaluza 2.1 Flow Analysis Software.

**Immunohistochemistry / Immunofluorescence**

Tumor tissues were harvested and fixed for 48 to 72 hours in 4% paraformaldehyde (Boston Bioproducts) and stored in 70% ethanol until further processing. Paraffin-embedded tumor sections (10 μm) were deparaffinized followed heat-mediated antigen retrieval for 30 min in IHC-Tek epitope retrieval solution (IHC World). After antigen retrieval, tumor sections were permeabilized with 100% methanol. Sections were blocked for 30 min with tris-NaCl (TNB) blocking buffer (PerkinElmer) and then incubated with rabbit anti-vaccinia virus antibody (Abcam) diluted 1:100 in TNB blocking buffer overnight in a humidified chamber at 4 °C. After incubation, tumor sections were washed and incubated with Alexa Fluor 488-conjugated goat anti-rabbit secondary antibody (Abcam, Grand Island, NY) for 1 hour at room temperature. Nuclei were counterstained with DAPI. Deparaffinized tumor sections (10 μm) were stained with mouse anti-human CD3 (Abcam, Grand Island, NY) and rabbit anti-human CD8α (Abcam, Grand Island, NY). Images were obtained using the 3DHISTECH Panoramic digital slide scanner and the associated Case Viewer software (3DHISTECH). For CD8 quantification after immunohistochemistry (IHC) staining, ImageJ (NIH) analysis was performed as per the standard recommended algorithm [2, 3]. The same method detected CCL5 (Human CCL5/RANTES Antibody (R&D, Minnesota)), CXCL10 (Rabbit Anti-CXCL10/IP10 antibody (Bioss, China)), Granzyme B (Human Granzyme B Antibody (R&D, Minnesota)) levels in the TME.

**Statistical analysis**

GraphPad Prism 8.0 software was used to construct all graphs and calculate statistical significance. Kaluza Analysis 2.1 software was used for FCM analysis and to generate plots. For two sets of fold-change measurements, a one sample t-test was used. For comparison of three or more sets of unpaired measurements, one-way ANOVA was performed with Tukey’s post-hoc test if all sets were analyzed, or Sidak’s post-hoc test if selected relevant pairs were analyzed. Significance from Kaplan-Meier survival curves were calculated with the Log-Rank test. P<0.05 was considered to be statistically significant.

**Supplementary references**

**References**

1. Guo Z, Shen B, Wu L. A humanized CD70 antibody LD70 and its preparation method and application.; 2018. p 12.

2. Crowe AR, Yue W. Semi-quantitative Determination of Protein Expression using Immunohistochemistry Staining and Analysis: An Integrated Protocol. Bio Protoc. 2019;9.

3. Park AK, Fong Y, Kim S, et al. Effective combination immunotherapy using oncolytic viruses to deliver CAR targets to solid tumors. Sci Transl Med. 2020;12:z1863.
